# Self-efficacy among patients with hemodialysis during the COVID-19 pandemic

**DOI:** 10.25122/jml-2021-0405

**Published:** 2022-06

**Authors:** Shereen Ahmed Qalawa, Sheren Ibrahim Eltahry, Azza Anwar Aly

**Affiliations:** 1 Department of Medical Surgical Nursing, College of Nursing, Qassim University, Al-Qassim, Kingdom of Saudi Arabia; 2 Department of Medical Surgical Nursing, Faculty of Nursing, Port-Said University, Port-Said, Egypt; 3 Department of Medical Surgical Nursing, Faculty of Nursing, Damanhour University, Damanhour, Egypt

**Keywords:** self-efficacy, patients, hemodialysis, comparative study, QOL – Quality of life, HD – Hemodialysis, ESKD – End-stage kidney diseases, DM – Diabetes Mellitus, KSA – Kingdom of Saudi Arabia, HRQOL – Health-related quality of life

## Abstract

Patients with hemodialysis face various problems with self-care behavior. Therefore, these patients need to control and manage adherence to enhance self-care, especially during the COVID-19 pandemic, which has particularly high morbidity in the elderly and co-morbid peoples. Self-efficacy was identified as a number of psychological factors that improve adherence and treatment outcomes among hemodialysis patients. This study aimed to investigate self-efficacy among patients with hemodialysis during the COVID-19 pandemic. A comparative descriptive research design was used to conduct the study. A convenience sample of 95 hemodialysis patients (male and females) was selected from Port-Said city, Egypt, and 115 hemodialysis patients (male and females) were selected from Saudi Arabia. Data collection included a structured interview with three parts assessing socio-demographic characteristics, anthropometric measurements, and self-efficacy. This study revealed a statistically significant difference between Egyptian and Saudi hemodialysis patients, mainly in the main score and levels of self-efficacy (p=0.001). There is a significant relationship between socio-demographic characteristics and chronic disease self-efficiency among Egyptian hemodialysis patients in terms of sex, age, marital status, work, and level of education.

## INTRODUCTION

Hemodialysis is the most common treatment for advanced and permanent kidney failure. Nowadays, a large number of people suffer from kidney disease and have multiple problems in terms of medical and treatment costs in health organizations all over the world [1]. COVID-19 is a major human threat that has become a pandemic; this new coronavirus has particularly high morbidity in the elderly and co-morbid people. The spread of the virus has increased, accompanied by widespread anxiety, fear, and uncertainty. Patients undergoing hemodialysis combine an inherent fragility and widespread problems of co-morbidities, and the specific setting in which many patients are treated frequently are dialysis units [2, 3].

Moreover, there is a relationship between fear and commitment shaped by self-efficacy, with higher levels of self-efficacy predicting greater commitment regardless of fear levels [4]. As a result, recognizing stressors, self-efficacy, and coping strategies among hemodialysis patients may help nurses and health care providers clearly understand patients' conditions [5]. Hemodialysis patients face various issues in self-care behavior and need to control and manage adherence to enhance their self-care. Self-efficacy was described as a number of psychological factors that improve adherence and treatment outcomes among hemodialysis patients [6]. Self-care is a vital component of managing chronic disease, and poor self-care of patients receiving dialysis is associated with an increased risk of death and hospitalization [7]. Hemodialysis can reduce patients' self-efficacy, affecting their confidence in managing their environment and life events. In addition, patients undergoing hemodialysis are more prone to psychological problems such as depression and low self-efficacy, and patients with depression are more likely to have low self-efficacy [8].

Furthermore, dealing with COVID-19 is a major risk factor for mental distress not only among patients but also among health workers [9]. There is increasing appreciation that self-efficacy of self-care in chronically ill patients is connected with improved quality of life (QOL). So, quality of life and self-efficacy can play a significant role in chronic kidney disease and treatment results [10, 11]. For example, spiritual therapy can be utilized as a successful intervention to improve spiritual well-being, self-esteem, and self-efficacy in hemodialysis patients [12]. Therefore, self-efficacy is an important issue to assess, and it is imperative to have protective procedures in place to plan preventive and protective strategies for future pandemics.

Furthermore, individual efficacy in minimizing negative emotions and ideas may be a stress-reduction approach [9]. COVID-19 has spread swiftly and widely since its outbreak in December 2019, putting the population under much psychological strain. Patients receiving hemodialysis (HD) experienced more severe trauma-related stress symptoms. When major public health events occur, dialysis patients, particularly HD patients, should receive rigorous psychiatric evaluation and adequate psychological support [13]. Global hemodialysis (HD) addresses the most common type of renal substitution treatment. Many examinations showed a solid connection between HD treatment and clinical results. The study was performed on 100 patients in the Hemodialysis Unit at Tanta College Medical Clinic, Egypt. Information was collected to investigate factors that affected hemodialysis as clinical factors, dialysis, lab, and radiological information, and uncovered lacking HD in 60% of cases [14]. The prevalence of end-stage kidney diseases (ESKD) on regular hemodialysis in Menoufia governorate in Egypt is gradually increasing from what has been previously reported, especially among elderly patients, with high blood pressure and diabetes mellitus (DM) being the most common causes of ESKD [15].

Furthermore, the conceptual framework highlighting that health promotion during lifestyle enhancement may be a vital subject material has received significant attention from the scientific community worldwide. The Pender model focuses on three areas of health promotion: individual characteristics and experiences, perception, specific behavioral effects, and behavioral outcomes [16]. Therefore, this study aimed to explore self-efficacy among patients with hemodialysis during the COVID-19 pandemic by assessing self-efficacy and the relationship with socio-demographic characteristics.

## MATERIAL AND METHODS

A comparative descriptive research design was used to conduct the current study. The study included adult males and females and was conducted in the renal dialysis units at Eltadamon Hospital, Al Salam General Hospital, and the assigned places for hemodialysis in Port-Said City, Egypt, and Aljadaani Hospital (KSA), from March 2021 to August 2021.

We included a convenience sample of all available patients undergoing hemodialysis during data collection. Patients were selected according to pre-established sample selection criteria. Patients who fulfilled the selection criteria were asked to participate in the study. We selected 95 hemodialysis patients (males and females) from Port-Said, Egypt, and 115 hemodialysis patients (male and female) from Saudi Arabia.

### Data collection

An Arabic structured interview questionnaire was developed by the researcher after an extensive literature review [17], intended to measure self-efficacy among hemodialysis patients. The questionnaire consisted of 3 parts: socio-demographics (age, occupation etc), anthropometric measurements (weight and height), and a self-efficacy assessment sheet which included 19 adapted self-efficacy items used to assess self-efficacy among hemodialysis patients. Items were scored on a Likert scale ranging from low (<50%), moderate (50–75%), and high (≥75%). The reliability was assessed using Cronbach alpha test=0.094

### Statistical analysis

The data was analyzed using the IBM SPSS version 20.0 software package. The qualitative data were expressed as percentages and numbers. Kolmogorov-Smirnov test was used to check the normality of distribution. The quantitative data were expressed by the range of the minimum and maximum, the mean, and the standard deviation. The Chi-square test was used for categorical variables to compare the different groups. Fisher's Exact or Monte Carlo correction for Chi-square correction was used when more than 20% of cells expected the number to be less than 5. Mann Whitney was used for quantitative variables distributed abnormally and to compare the two study groups. Statistical significance was considered at P-value<0.05 and highly significant at P-value<0.00.

## RESULTS

Table 1 shows a significant difference between dialysis patients in Egypt and Saudi Arabia regarding socio-demographic items related to gender, marital status, residence, job, income, and educational level (p=0.039, 0.001, 0.009, respectively).

**Table 1:** Comparison between samples according to socio-demographic data (n=210).

Socio-demographic data	Egypt (n=95)		Saudi (n=115)		χ2	p
	No.	%	No.	%		
**Sex**						
Male	46	48.4	72	62.6	4.254*	0.039*
Female	49	51.6	43	37.4		
**Age (years)**						
20–30	8	8.4	9	7.8	6.321	0.097
21–40	12	12.6	26	22.6		
41–50	34	35.8	47	40.9		
Above 51	41	43.2	33	28.7		
**Marital Status**						
Married	11	11.6	54	47.0	40.020*	<0.001*
Divorced	56	58.9	44	38.3		
Absolute	8	8.4	12	10.4		
Widowed	20	21.1	5	4.3		
**Resides**						
The wife	48	50.5	18	15.7	30.069*	<0.001*
Sons	17	17.9	28	24.3		
Relatives	13	13.7	29	25.2		
Alone	17	17.9	40	34.8		
**Job**						
Does not work	31	32.6	26	22.6	34.296*	<0.001*
Works	42	44.2	88	76.5		
Pension	22	23.2	1	0.9		
**Income level**						
Enough	30	31.6	72	62.6	20.053*	<0.001*
Not enough	65	68.4	43	37.4		
**Educational level**						
Illiterate	9	9.5	15	13.0	15.323*	0.009*
Primary education	11	11.6	12	10.4		
Intermediate education	17	17.9	42	36.5		
High school education	39	41.1	24	20.9		
University education	15	15.8	20	17.4		
Postgraduate	4	4.2	2	1.7		

χ2 – Chi-square test; * – Statistically significant at p≤0.05.

There was a significant difference between dialysis patients in Egypt and Saudi Arabia regarding medical history related to the duration of hemodialysis, weekly dialysis time, and other diseases (p=0.001) (Table 2). Furthermore, there was a significant difference in the anthropometric measurements of height (p=0.001) between Egyptian and Saudi hemodialysis patients (Table 3). In addition, there was a significant difference in the main score and levels of self-efficacy (p=0.001) between Egyptian and Saudi hemodialysis patients (Table 4).

**Table 2:** Comparison between the two samples according to medical history (n=210).

Items	Egypt (n=95)		Saudi (n=115)		χ2	p
	No.	%	No.	%		
**Since when do you suffer from kidney failure?**						
less than two years	26	27.4	69	60.0	25.235*	^MC^p <0.001*
2–5 years old	34	35.8	29	25.2		
More than 5 years	34	35.8	16	13.9		
I do not remember	1	1.1	1	0.9		
**How many times do you do dialysis per week?**						
Once	3	3.2	0	0.0	33.531	^MC^p <0.001*
Twice	50	52.6	64	55.7		
Three times	42	44.2	28	24.3		
Irregular (as per doctor's orders)	0	0.0	23	20.0		
**Do you have anyone in your family who has kidney failure?**						
No	41	43.2	90	78.3	34.195*	<0.001*
Yes	40	42.1	25	21.7		
I do not know	14	14.7	0	0.0		
**Do you have any other of the following diseases?**						
Diabetes Mellitus	51	53.7	0	0.0	81.539*	<0.001*
Hypertension	80	84.2	0	0.0	156.437*	<0.001*
Cancer	6	6.3	0	0.0	7.477*	^FE^p=0.008*
Depression	10	10.5	2	1.7	9.654*	^FE^p=0.002*
Deep vein thrombosis	14	14.7	3	2.6	14.051*	^FE^p=0.001*
Heart disease	31	32.6	12	10.4	20.029*	^FE^p<0.001*
Heart attack	22	23.2	10	8.7	12.215*	^FE^p=0.001*
Coronary artery disease	59	62.1	3	2.6	103.627*	^FE^p<0.001*
Systemic Lupus	4	4.2	5	4.3	3.321	^FE^p=0.207
Rheumatic heart diseases	4	4.2	4	3.5	9.220*	^FE^p=0.005*
Cholesterol increase	61	64.2	21	18.3	61.649*	^FE^p<0.001*
Chest crunch	12	12.6	9	7.8	10.801*	^FE^p=0.003*
Tuberculosis	12	12.6	10	8.7	10.264*	^FE^p=0.004*
Osteoporosis	32	33.7	2	1.7	51.387*	^FE^p<0.001*
Cataract	16	16.8	14	12.2	12.054*	^FE^p=0.002*
Glaucoma	10	10.5	7	6.1	10.791*	^FE^p=0.003*
Stomach ulcer	14	14.7	13	11.3	9.929*	^FE^p=0.004*

χ2 – Chi-square test; MC – Monte Carlo; ^FE^ – Fisher Exact; * – Statistically significant at p≤0.05.

**Table 3:** Comparison between the two samples according to their anthropometric measurements (n=210).

Items	Egypt (n=95)		Saudi (n=115)		χ2	p
	No.	%	No.	%		
**Height (cm)**						
<150	14	14.7	52	45.2	24.303*	<0.001*
151–175	59	62.1	52	45.2		
176–200	22	23.2	11	9.6		
**Weight (kg)**						
<50	3	3.2	9	7.8	2.882	0.410
50–75	36	37.9	48	41.7		
76–100	42	44.2	44	38.3		
>100	14	14.7	14	12.2		

χ2 – Chi-square test; * – Statistically significant at p≤0.05.

**Table 4:** Comparison between total & mean scores of chronic diseases self-efficacy scale among subjects (n=210).

Chronic disease self-efficacy scale	Egypt (n=95)		Saudi (n=115)		Test of sig	p
	No.	%	No.	%		
Low (<50%)	60	63.2	1	0.9	χ2=117.955*	<0.001*
Moderate (50–75%)	33	34.7	50	43.5		
High (≥75%)	2	2.1	64	55.7		
**Total Score**						
Min–Max	0.0–48.0		26.0–57.0		U=512.0*	<0.001*
Mean±SD	21.23±12.12		42.98±4.92			
Median	23.0		44.0			
**Average score**						
Min–Max	0.0–2.53		1.37–3.0		U=512.0*	<0.001*
Mean±SD	1.12±0.64		2.26±0.26			
Median	1.21		2.32			
**% score**						
Min–Max	0.0–84.21		45.61–100.0		U=512.0*	<0.001*
Mean±SD	37.25±21.27		75.41±8.63			
Median	40.35		77.19			

U – Mann Whitney test; χ2 – Chi-square test; * – Statistically significant at p≤0.05.

Table 5 shows a significant relationship between socio-demographic characteristics and chronic disease self-efficacy among Egyptian hemodialysis patients, mainly in terms of gender, age, marital status, job, and level of education (p=0.041, 0.001, 0.025, 0.001, 0.005). In contrast, there was no significant relationship between socio-demographic characteristics and chronic disease self-efficacy among Saudi hemodialysis patients.

**Table 5:** The relationship between chronic diseases self-efficacy scale and hemodialysis patient's socio-demographic data (n=210).

Socio-demographic data	Chronic disease self-efficacy scale											
	Egypt (n=95)						Saudi (n=115)					
	Low (n=60)		Moderate (n=33)		High (n=2)		Low (n=1)		Moderate (n=50)		High (n=64)	
	No.	%	No.	%	No.	%	No.	%	No.	%	No.	%
**Sex**												
Male	24	40.0	20	60.6	2	100.0	0	0.0	29	58.0	43	67.2
Female	36	60.0	13	39.4	0	0.0	1	100.0	21	42.0	21	32.8
**χ2(^MC^p)**	5.381*(0.041*)						**2.607 (0.231)**					
**Age (years)**												
20–30	2	3.3	5	15.2	1	50.0	0	0.0	5	10.0	4	6.3
21–40	3	5.0	8	24.2	1	50.0	1	100.0	8	16.0	17	26.6
41–50	19	31.7	15	45.5	0	0.0	0	0.0	23	46.0	24	37.5
Above 51	36	60.0	5	15.2	0	0.0	0	0.0	14	28.0	19	29.7
**χ2(^MC^p)**	28.316*(<0.001*)						**6.131 (0.426)**					
**Marital Status**												
Married	3	5.0	7	21.2	1	50.0	0	0.0	23	46.0	31	48.4
Divorced	36	60.0	19	57.6	1	50.0	1	100.0	17	34.0	26	40.6
Absolute	4	6.7	4	12.1	0	0.0	0	0.0	5	10.0	7	10.9
Widowed	17	28.3	3	9.1	0	0.0	0	0.0	5	10.0	0	0.0
**χ2(^MC^p)**	12.563*(0.025*)						**10.153 (0.109)**					
**Resides**												
The wife	30	50.0	17	51.5	1	50.0	0	0.0	4	8.0	14	21.9
Sons	14	23.3	3	9.1	0	0.0	0	0.0	16	32.0	12	18.8
Relatives	4	6.7	8	24.2	1	50.0	0	0.0	13	26.0	16	25.0
Alone	12	20.0	5	15.2	0	0.0	1	100.0	17	34.0	22	34.4
**χ2(^MC^p)**	**9.822 (0.078)**						**7.755 (0.183)**					
**Job**												
Does not work	25	41.7	6	18.2	0	0.0	0	0.0	13	26.0	13	20.3
Works	17	28.3	23	69.7	2	100.0	1	100.0	36	72.0	51	79.7
Pension	18	30.0	4	12.1	0	0.0	0	0.0	1	2.0	0	.0
**χ2(^MC^p)**	16.117*(<0.001*)						**5.237 (0.566)**					
**Income level**												
Enough	15	25.0	14	42.4	1	50.0	1	100.0	27	54.0	44	68.8
Not enough	45	75.0	19	57.6	1	50.0	0	0.0	23	46.0	20	31.3
**χ2(^MC^p)**	**3.644 (0.170)**						**3.140 (0.164)**					
**Educational level**												
Illiterate	9	15.0	0	0.0	0	0.0	0	0.0	8	16.0	7	10.9
Primary education	10	16.7	1	3.0	0	0.0	0	0.0	6	12.0	6	9.4
Intermediate education	12	20.0	5	15.2	0	0.0	0	0.0	16	32.0	26	40.6
High school education	20	33.3	17	51.5	2	100.0	0	0.0	11	22.0	13	20.3
University education	9	15.0	6	18.2	0	0.0	0	0.0	9	18.0	11	17.2
Postgraduate	0	0.0	4	12.1	0	0.0	1	100.0	0	0.0	1	1.6
**χ2(^MC^p)**	20.634*(0.005*)						**13.179 (0.249)**					

χ2 – Chi-square test; MC – Monte Carlo; * – Statistically significant at p≤0.05.

Finally, there was a significant relationship between chronic disease self-efficacy and medical history among Egyptian hemodialysis patients, mainly in items of the duration of dialysis and weekly dialysis time (p=0.001, 0.018) (Table 6). However, among Saudi hemodialysis patients, there was a significant relationship only in terms of the duration of dialysis (p=0.046)

**Table 6:** The relationship between chronic diseases self-efficacy scale and patient's medical and family history (n=210).

Dialysis data	Chronic disease self-efficacy scale											
	Egypt (n=95)						Saudi (n=115)					
	Low (n=60)		Moderate (n=33)		High (n=2)		Low (n=1)		Moderate (n=50)		High (n=64)	
	No.	%	No.	%	No.	%	No.	%	No.	%	No.	%
**Since when do you suffer from kidney failure?**												
Less than two years	8	13.3	16	48.5	2	100.0	0	0.0	29	58.0	40	62.5
2–5 years old	21	35.0	13	39.4	0	0.0	0	0.0	13	26.0	16	25.0
More than 5 years	31	51.7	3	9.1	0	0.0	0	0.0	8	16.0	8	12.5
I do not remember	0	0.0	1	3.0	0	0.0	1	100.0	0	0.0	0	0.0
**χ2(^MC^p)**	29.195*(<0.001*)						12.616*(0.046*)					
**How many times do you do dialysis per week?**												
Once	1	1.7	2	6.1	0	0.0	0	0.0	0	0.0	0	0.0
Twice	26	43.3	23	69.7	1	50.0	1	100.0	29	58.0	34	53.1
Three times	33	55.0	8	24.2	1	50.0	0	0.0	9	18.0	19	29.7
Irregular (as per doctor's orders)	0	0.0	0	0.0	0	0.0	0	0.0	12	24.0	11	17.2
**χ2(^MC^p)**	10.252*(0.018*)						**3.449 (0.526)**					
**Do you have anyone in your family who has kidney failure?**												
No	28	46.7	12	36.4	1	50.0	1	100.0	38	76.0	51	79.7
Yes	24	40.0	16	48.5	0	0.0	0	0.0	12	24.0	13	20.3
I do not know	8	13.3	5	15.2	1	50.0	0	0.0	0	0.0	0	0.0
**χ2(^MC^p)**	**3.666 (0.437)**						**0.689 (0.731)**					

χ2 – Chi-square test; MC – Monte Carlo; * – Statistically significant at p≤0.05.

## DISCUSSION

Hemodialysis can lead to significant changes in the physical and psychological status, daily living activities, and marital and social life status. However, assessing health-related quality of life (HRQOL) helps plan individual treatment strategies and determines the effectiveness and quality of medical and social care provided [18]. Regarding socio-demographic characteristics of the studied patients (Table 1), our study showed a significant difference between Egyptian and Saudi dialysis patients mainly in socio-demographic items related to gender, marital status, residence, job, income, and educational level. In what concerns hemodialysis patients and medical history (Table 2), the study revealed a significant difference between Egyptian and Saudi patients with hemodialysis, mainly in medical history items related to the duration of hemodialysis, weekly dialysis time, and other diseases with a family history of renal failure.

This finding agrees with another study from Egypt [18], which highlighted the primary essential clinical disorders affecting QOL that indirectly affected patients' self-efficacy: hepatitis C virus infection, anemia, DM, and sleep disturbances. However, increasing patients' education about hemodialysis and their caregivers regarding problems associated with hemodialysis treatment is very important to decrease the patients' problems and improve their self-efficacy and QOL. The current study showed a significant difference between Egyptian and Saudi hemodialysis patients only in terms of height. This finding agrees with another study [19] that revealed the importance of attention to height differences in clinical and laboratory characteristics that subsist in Egyptian HD patients and should be considered when developing treatment and care guidelines to ensemble the gender-related variations.

Regarding the chronic disease self-efficacy scale, there was a significant difference between Egyptian and Saudi hemodialysis patients, mainly in the main score and levels of self-efficacy. These findings support other studies from Egypt [20] that highlighted the importance of patients' level of self-efficacy, self-care behavior, and physical ability to control their disease and symptoms. Individuals with sufficient self-care and self-efficacy can effectively meet their self-care requirements, assume responsibility for their own health, and plan and organize their daily activities. In contrast, in Egypt, [5] there were statistically significant negative correlations between total scores of coping strategies, self-efficacy, and stressors among hemodialysis patients. At the same time, there was a positive, statistically significant correlation between the total scores of coping strategies and self-efficacy.

Furthermore, another study reported that family support, fatigue, and monthly income are significant criteria that affect the hemodialysis patients' quality of life [21]. Additionally, Ramezani et al., 2019 [6] highlighted the benefits of self-efficacy, and nurses must employ educational programs based on the theory of self-efficacy to improve self-care behaviors among patients with dialysis.

In Iran, the self-efficacy attributes of the family center empowerment model signify dimensions of self-efficacy for individuals with chronic diseases that can be used to increase and perform programs to empower chronic disease patients [22]. Another study [16] recommended implementing an educational program for patients undergoing hemodialysis to gain the positive effects of this program in supporting the well-being of those patients. Moreover, Lee and Noh [7] proposed that educational strategies to develop self-efficacy, health motivation, and knowledge must be integrated into self-care programs, including their family members, to enhance self-care. There was a significant relationship between socio-demographic characteristics and chronic disease self-efficacy among Egyptian hemodialysis patients, mainly in sex, age, marital status, job, and level of education. In contrast, there was no significant relationship between socio-demographic characteristics and chronic disease self-efficacy among Saudi hemodialysis patients. This finding agrees with another study [18] which identified that self-efficacy is linked with the quality of life among hemodialysis patients. It is imperative to investigate socio-demographic factors affecting HRQOL and indirectly self-efficacy, such as age, sex, education, occupation, and marital status.

This study revealed a statistically significant relationship between chronic disease self-efficacy and medical history among Egyptian hemodialysis patients in the duration of suffered dialysis and weekly dialysis time, while for Saudi hemodialysis patients, only in the duration of suffered dialysis. This finding is consistent with the findings of Mohamed et al. [4], who revealed that compared with the control group, the total average scores of the study group for patients' knowledge, using the Nottingham scale, revealed statistically significant differences.

They concluded that developing a self-care plan could effectively improve patients' performance and dependence on hemodialysis. Finally, Aziz et al., 2019 [8] reported that self-efficacy among hemodialysis patients is more impaired than those with other medical conditions such as diabetes, hypertension, and chronic lung disease. Another study [23] emphasized the importance of empowering hemodialysis patients and their caregivers, who can support them in managing health-related issues and develop their self-efficacy, and recommend this before starting dialysis treatment.

Health professionals should educate patients according to their needs and their family and implement empowerment programs to arrange them for the activities needed. Therefore, educational intervention for behavior change based on self-efficacy theory can effectively improve self-care behavior to promote self-care of hemodialysis patients [6].

## CONCLUSION

The current study concluded that there is a significant difference between dialysis patients in Egypt and Saudi Arabia, mainly in the main score and levels of self-efficacy. In addition, a significant relationship was found between socio-demographic characteristics and chronic disease self-efficacy among Egyptian hemodialysis patients in sex, age, marital status, job, and level of education. In contrast, there was no significant relationship between socio-demographic characteristics and chronic disease self-efficacy among Saudi hemodialysis patients. Based on the results, it is recommended to strengthen the instructional scheme for hemodialysis and its management to increase self-efficacy among those patients.
